# What do parents think of using informational videos to support recruitment for parenting trials? A qualitative study

**DOI:** 10.1186/s13063-021-05826-0

**Published:** 2021-12-04

**Authors:** Maiken Pontoppidan, Sarah Blower, Julie Nygaard Solvang, Tracey Bywater

**Affiliations:** 1 VIVE – the Danish Centre for Social Science Research, Herluf Trolles Gade 11, 1052 Copenhagen, Denmark; 2 Department of Health Sciences, University of York, Area 2 Seebohm Rowntree Building, Heslington, York, YO10 5DD UK

**Keywords:** Parents, Recruitment, Trial, Informed consent, Informational video, Qualitative

## Abstract

**Background:**

Lower than expected recruitment and retention rates are common challenges in parenting trials—particularly for community-based trials targeting parents of young children that rely on face-to-face recruitment by frontline workers. Recruitment requires parental informed consent, yet information sheets have been criticized for being lengthy and complex, and particularly challenging for parents with low literacy. Recent innovations include ‘talking head’ information videos. This paper aims to explore parent perceptions of using a ‘talking head’ video to support informed consent, recruitment, and retention procedures in parenting trials.

**Methods:**

We conducted semi-structured interviews with a sample of 24 mothers recruited after their final follow-ups in two different parenting trials in Denmark. Before consenting to participate in the trials, parents were invited to view a video of a member of the study team giving information about the study, and again before the interviews for the current study. The audio data was transcribed and thematic analysis was conducted.

**Results:**

We identified three overarching themes: (1) general impression of the video, (2) thoughts on participation in research, and (3) recruitment and retention. Participants were generally positive in their appraisal of the two talking head informational videos. We found that participants felt that a mix of paper-based and video-based sources of information would enable them to make an informed choice about whether to participate in a research study. We also found that a professionally produced video featuring a key member of the study team produced a feeling of commitment to the study that could impact retention rates.

**Conclusions:**

Informational videos are acceptable to parents; however, co-production or participant/patient involvement in the development of such videos is recommended. Informational videos may not increase recruitment but have the potential for improving retention. Key design recommendations are to ensure a ‘professional’ look to the video, to supplement videos with paper-based information, to keep the length to < 3 min, and for the ‘talking head’ part to feature a key member of the study team.

**Supplementary Information:**

The online version contains supplementary material available at 10.1186/s13063-021-05826-0.

## Background

Although randomized controlled trials are the ‘gold standard’ for testing the effectiveness of parenting programmes in real-life settings, recruitment and retention of participants can be problematic and act as major barriers to successful completion of a trial; for instance, only a small proportion of UK trials successfully recruit to time and target [1–4]. There is a dearth of rigorous evidence about the most effective strategies for supporting research teams in the recruitment and retention of participants [5]. This is a major issue as poor recruitment or retention may lead to inconclusive results and issues around generalizability of findings [6]. Delays caused by recruitment difficulties can also reduce the impact of research with less power, and without effective recruitment into parenting trials, our collective goal to identify the most effective ways to improve the wellbeing of children and families will be difficult to achieve [5, 7].

Parent refusal to consent to participate is a key barrier for successful recruitment targets of trials involving children [8]. There is, however, little research on how parents (or children) make decisions about whether or not to participate in research trials, what information is important to them, and in what format [9–11]. Traditional participant information sheets (PIS) have been criticized for being too long and difficult to understand [12–17], with the focus guided by regulatory agencies, and not what parents want, or need, to know about [12]. Studies have demonstrated that a substantial proportion of trial participants do not always understand or remember information from PIS [18–21]. This is alarming from an ethical perspective and does not lend itself to inclusivity or equity as participants with low levels of literacy or whose language is different to that in which the information is presented are at increased risk of not fully understanding what they are consenting to [22].

Trials of real-world parenting programmes delivered in community settings oftentimes rely on frontline staff (such as health visitors and family support workers) to identify and, in some cases, recruit participants during routine contacts [23, 24]. Person-to-person engagement strategies are effective but require significant resources, e.g., on training and time used [25]. Informational videos are potentially less resource-intensive when used as a tool to support practitioners in their conversations with parents about a trial, and what participation will entail.

Although modern technology can support the quick and inexpensive production of videos, costs could quickly escalate if relying on a filmmaker or animation company. Once produced, the video has the potential for a wide reach [25]. Costs would be needed to be weighed up in each potential trial to ensure that the sample size/size of trial can accommodate these costs and if the additional work is worth the potential return of increased participation and retention. Informational videos offer several advantages over traditional paper consent forms [22]. One study examining the effect of using four different video-based approaches to engage parents in intervention studies found that all four video approaches increased parents’ interest in participating in the study, but only the most streamlined version of the video significantly increased enrolment compared to control conditions [25]. Another study examining the effect of adding video clips to standard PIS in a parenting trial found that the video clips did not increase recruitment into the trial [26]. However, the impact of informational videos on trial recruitment and retention rates requires further exploration given the lack of research in this area.

Informational videos may be easier to understand and more user-friendly than the traditional, and most likely more lengthy, PIS, although there is a recent move towards shorter PIS [27].

This paper aims to explore parent perceptions of using a ‘talking head’ video to explain the background, aim, and process of the study to participants in two studies undertaken in Denmark. Specifically, we examine if ‘talking head videos’ are acceptable and useful as a replacement, or supplement to, written participant information sheets and whether parents think that informational videos could increase engagement and retention to a trial.

## Methods

We undertook a qualitative telephone interview study with 24 parents in Denmark who participated in one of two parenting trials. The qualitative study was conducted after the parents had received the interventions and all outcomes were collected.

### Participants

Participants were recruited from two separate randomized controlled trials of parenting interventions: (1) the Incredible Years Parents and Babies (IYPB) trial (2013–2015) and (2) the Parent Management Training Oregon (PMTO) trial (2013–2016). The IYPB trial was aimed at a universal sample of parents of infants whereas the PMTO trial was aimed at a more clinical group of parents of school-aged children with behaviour problems. In both trials, frontline workers were responsible for recruiting parents. All parents received a traditional PIS leaflet with information about the study from the frontline worker. The information in the leaflets was relatively easy to read and included pictures, names, and contact information of the principal investigator (PI) and a second member of the research team. We used relatively similar talking head information videos to support recruitment in both studies, and it was voluntary to watch the video. The videos were included in the recruitment process for the following reasons: (1) to increase the probability of front workers providing families with the correct information about the trial and (2) to ensure that families received and understood the information. Both mothers and fathers were invited to participate in both trials, but in most cases, it was the mother’s email or mobile number that was available. As the qualitative study on recruitment was not a part of the original ethics approval, we obtained supplementary ethics approval from the internal review board at SFI—the Danish National Center for Social Research (now VIVE - The Danish Center for Social Science Research). Only mothers accepted the invitation to participate in the interviews. All interviews were conducted in Danish in the period February to May 2017.

#### IYPB trial

The IYPB pilot trial examined the effect of the IYPB programme offered to a universal group of 112 parents of infants aged 0–4 months [28–30]. Health visitors recruited the families and notified SFI about the family. An interviewer then went to visit the family to obtain written consent and baseline assessment. The health visitors were instructed to give the PIS to the family, tell the families about the video, and show it to the family, if the family wished to see the video. Both health visitors and the interviewer gave the family the information leaflet that included a link to the video and a QR code. Then, SFI randomized the family and sent a notice about the allocation to the municipality. Participants who consented to participate in the IYPB intervention trial and who gave consent to be sent newsletters by email were informed of the extra interview about the recruitment video in a newsletter. Parents who wanted to participate sent their contact information and were subsequently contacted by the research staff. We interviewed 14 mothers from the IYPB trial.

#### PMTO trial

The PMTO trial examined the effect of PMTO offered to 128 parents of children aged 3–13 years with behaviour problems [31, 32]. Eligible participants were families referred to municipal treatment because of child behavioural problems. Case officers or pre-admission assessment committees in the municipality recruited the families to the trial. The practitioner who recruited the family to the project gave the family the PIS leaflet that included a link to the video and a QR code. SFI randomized families to either PMTO intervention or service as usual following consent to participate in the study. We contacted a random sample of 20 PMTO participants by text message and informed them about the extra interview on the recruitment video. Parents who wanted to participate responded to the text message and were subsequently contacted by the research staff. We interviewed 10 mothers from the PMTO trial.

### Procedure

We conducted the interviews via telephone. Telephone interviews are commonly used in survey research, but can also be used in smaller qualitative studies as it is less time consuming and flexible than face-to-face interview methods [33, 34]. When originally recruited, all participants received information on how to access the video. Few of the participants interviewed for this study, however, remember watching the video at the time they were recruited to the trial. We, therefore, asked the participants to watch the video before the interview. Participants could access the video through a link they received through a direct email. As the mothers did not remember having seen the video before recruitment, the interviewer asked the participants to try to imagine how they would feel if they had watched the video before deciding whether to participate in the study or not (what-if-questions). The answers are therefore hypothetical as the participants are thinking about what they would have done, not necessarily what they did.

### Videos

The communication department at SFI produced a short video for each trial. Both videos were recorded in the same room and followed the same structure. The IYPB trial video lasted 2:45 min and featured the PI, whereas the PMTO trial video lasted 2:13 min and featured the head of the department who was responsible for both trials. The visuals and audio were further supported by text on the screen to highlight the important messages. The videos could be accessed on the SFI homepage when the trials were running. This homepage no longer exists but the videos can be accessed (in Danish) at https://www.youtube.com/watch?v=wgb99I6mDmY (IYPB) and https://www.youtube.com/watch?v=j7PXRg8w2mI&t=3s (PMTO). The videos were accessed 88 times (IYPB) and 150 times (PMTO) during the trial recruitment period, but we have no further information on who accessed the videos, or how many times an individual may have accessed the videos.

### Interviews

The 24 interviews were semi-structured and consisted of six overall questions with sub-questions and prompts. See Additional file 1 for the full interview guide. The question guide included questions on what the mother thought of the video (relevance, length), the pros and cons of using a video for recruitment, the impact of seeing the researcher behind the project in the video, and thoughts and concerns for participating in a research project. An experienced research assistant who had not been involved in either of the trials conducted all interviews. The interviews lasted from 8 to 20 min. At the end of the interview, all mothers were asked if they had anything else to add to make sure that they did not have anything further to add. None of the questions or responses contained personal information. All mothers received a 200 DKK (~ 27 EUR) gift card by email after the interview.

### Data analysis

All participants gave consent to record the interview with a voice recorder. The research assistant who conducted the interviews transcribed the interviews in full. Data files and transcriptions were stored on a safe drive and only researchers involved in the project had access to the files. MP and JS analysed the data according to thematic analysis [35]. We based the analysis on the specific research question and followed the recommended six phases: (1) familiarization with the data, (2) generating codes to identify important features, (3) generating themes, (4) reviewing themes, (5) defining and naming themes, and (6) writing up the analysis. MP and JS separately read the interview transcriptions and systematically applied codes to the interviews. We applied a semantic level approach to the coding and generated themes from the explicit meaning of the data. The 24 interviews provided us with sufficient and rich data, and we decided that no further interviews were needed. Data saturation was reached when no further themes could be generated. We conducted the analyses in NVivo 11 and translated selected quotes into English.

## Results

We interviewed 24 mothers who had participated in either of the two trials. All mothers were Danish except for one. Based on the coding of the interviews, we identified the following three overarching themes: (1) general impression of the video, (2) thoughts about participation in research, and (3) recruitment and retention. For each theme, we identified one or more sub-themes.

### Theme 1: General impression of the video

All mothers watched the video before the interview in the current study, but many of them did not recall watching the video before recruitment to the trial they participated in (IYPB or PMTO). Three mothers were either sure or almost certain they had seen the video as part of the recruitment process for the trial.

All participants expressed a positive general impression of the video and the information it provided. They found that the videos were informative and explanatory and that they contained useful information. A participant noted: “I think that it [the video] explains the things that you need to know before you agree to participate” [mother 2]. When asked about their general opinion of the videos, several of the participants expressed that it was “fine”—a word, which in Danish indicates a moderate positive and satisfactory attitude towards something, e.g. “It is short and describes what it is supposed to. I think that it’s fine” [mother 3]. Throughout the interviews, the mothers elaborated on this topic, highlighting both pros and cons of the recruitment videos.

The videos were perceived as a trustworthy source of information by the participants. This is exemplified by this quote: “It is more trust-based to see a video than to see a leaflet, that you don’t really know who wrote” [mother 8]. A few mothers pointed out that the video was a bit boring and not very exciting to watch. When asked about the length of the video, almost all participants (21) agreed that it was appropriate (2:45 min for the IYPB and 2:13 min for the PMTO). Only one mother felt that the video should be longer. A few participants pointed out that there was a risk of losing focus if the video was longer, and six mothers stated that the video should not be longer. This is clearly expressed by mother 5 who said: “It should not be longer - then I think people would not like to see it”.

#### Advantages of using a video for recruitment

All participants expressed that they thought it would be beneficial to use video for recruitment. Most mothers provided several reasons for this. They felt that the video was catchy and enjoyed the visual inputs. This is exemplified by a mother who said that “the information is less dry compared to getting it in a leaflet” [mother 9]. They also highlighted the accessibility of the video. At a practical level, they liked that all information was easily accessible just with a click on a link or a QR code. At a more abstract level, they expressed that it was easy to comprehend the content and information in the video. Similarly, nine participants perceived the video as straightforward and easy to understand.

The mothers felt that using an informational video in the recruitment process had advantages over other formats. Some mothers felt that it was more time-efficient because it was faster to watch the video than reading the info leaflet and because they could do it at a time that was convenient for them, e.g. while doing the dishes. This comes clearly through in this quotation from mother 2: “Then there is the time factor in that it is faster and easier to watch a video. You usually have a busy schedule with children and work and all of that then it is easier and more manageable just to see a video than it is to have to read a lot of papers or something”.

Some mothers felt that a video is easier to remember than written information and one mother felt that the spoken word is harder to misunderstand than the written. She explained it this way: “When you read something, you sometimes read it the wrong way, while when it is spoken, at least I find it easier to hear what needs to be heard and not read something that was not there” [mother 8].

A point that several participants highlighted was that using a video was a clear advantage for participants with low literacy. This was expressed by both a mother with reading difficulties and a mother with attention deficit hyperactivity disorder (ADHD) but also by mothers who did not necessarily have low literacy themselves. By using a video, parents with low literacy can understand the information as easily as parents without literacy problems. The mother with ADHD expressed it this way: “It is easier to reach everyone [with a video] … it can sometimes be hard to write it in a way so that you reach those who are not so academically strong … you reach everyone no matter if you are a good or bad reader because everyone can understand what she is saying and it is a simple explanation which would be harder to write in a leaflet” [mother 7].

#### Disadvantages of using a video for recruitment

Although participants were generally positive, they also pointed out various disadvantages of the videos. Several mothers were concerned that participants would overlook the link to the video in the information leaflet and thereby not use the video. Another participant felt that a link sent in an email was easier lost than a physical leaflet that she could have on the desk. Although most participants found the length of the video appropriate, some expressed that the videos missed important information such as a description of the interventions they could be randomized to, and practical information about the process of the study. This is exemplified by mother 5 who said: “I maybe missed a bit more practical information in the video. You know, more information on what exactly what going to happen for instance that you received a questionnaire that you should fill out…”. One participant stated that she needed more information about how the research would be used.

Some of the participants commented on the video format and set-up. One found that it looked like a quick fix and was non-professional. For some participants, it was distracting that the editing of the video was not professional, that the room was disorderly, and that the audio did not correspond completely with the written text shown on the videos. Some of the interviewed mothers had suggestions of how to improve the video. Suggestions included distributing the video through text message or social media and including a presentation of the research institution and the goals of the study.

#### Video versus print

When comparing the video format to the written, several participants expressed a preference for the video format but for various reasons. Several pointed to the overwhelming amount of received information after the birth of the infant and the piles of leaflets and pamphlets that accumulate. They found it easier to watch a video with the relevant information. Again, they highlighted the accessibility in the comparison: “If you just leaf through [the pamphlet], then you may skip some things – here you get all the information in two minutes – everyone can do that. If you get a pamphlet you can easily just leave it” [mother 6]. The short and accessible format of the videos appealed to many of the participants. A few participants preferred to receive important information about a trial in written form. The majority of mothers agreed that the video should be a supplement to a written or printed PIS. Only one mother said that she would prefer seeing the video and not getting an information leaflet at all—especially if the video included clips with written information.

### Theme 2: Thoughts about participation in research

We asked all the mothers about their thoughts and concerns about participating in a randomized trial and their motivation for participating. It was clear from the interviews that several mothers from both studies did not remember that they had received the information leaflet when they were originally recruited for the study. We do not know whether they did not receive the information leaflet or if they do not remember receiving it. The interviews further showed that not all the participants had fully understood what the trials involved when they were recruited. One mother for instance did not understand that it was a research project: “We were not told directly that it was a research project – we were told it was to get some help for our daughter” [mother 14].

#### Randomization

In both trials, SFI randomized families to either intervention or care as usual. The randomization was clearly described in the information leaflet and the front personnel who recruited the families were instructed to inform all families about the randomization. Several mothers said that they were not aware that they had been randomized or did not feel that they had been informed about this: When the interviewer asked one of the mothers if she had any concerns about the fact that it was a random allocation she responded “I did not know” [mother 6]. One mother even said that she did not know if she would have agreed to participate if she had understood that it was the researchers who by random allocation decided whether the family would receive the intervention or not. When she was asked if she would have responded differently if she had understood that she would be randomized, she responded: “Then I wouldn’t have joined. And I know others that wouldn’t have either. But I did not think about it at that time, and I’m also happy [that I joined]” [mother 15]. Even though she did not fully understand the consequences of participating, she was happy that she did join the study. When asked if the video would have made it clearer for her, she said that it would. The participants agreed that the video was clear about the randomization. One mother said: “You are not in doubt that something will be chosen for you and the different possibilities there are” [mother 6].

#### Motivation

When asked about why they joined the study, most mothers expressed that they did it to contribute to the research and to help improve the conditions for parents in the future. The altruistic motivation is evident in this quotation: “If my answers and other participants’ answers could help make it better for the municipalities finding the right interventions [for new parents], find out what works and doesn’t work, then I’m happy to do that. That was actually my only thought; if it can help other [families]” [mother 9]. The mothers were generally positive about contributing to research and thereby improving circumstances for future parents in a similar situation. For many mothers, the fact that it was a research project was important. One mother said: “I think you should always support such projects” [mother 12]. Several mothers also said that they were interested in the results and wanted to learn from the study themselves. Answering questionnaires and being followed by researchers was for many mothers a positive experience. One of the mothers framed it like this when asked why she wanted to participate: “To be a part of a research project where you can learn about yourself and your children and what is happening in the new life” [mother 13]. We find the altruistic motivation to participate and the positive attitude toward being part of a research project in both the parents with infants (IYPB) and the parents with older children with behaviour problems (PMTO). Some mothers, however, did also see the intervention as a much-needed help for their own family and an opportunity to develop as a parent and were therefore motivated to participate.

### Theme 3: Recruitment and retention

#### Recruitment

Most of the participants agreed that seeing a recruitment video would not be critical for their motivation to participate in the trial. As they had decided to participate in the trial at the time they were interviewed for this study, they were motivated before watching it. Some mothers, however, expressed that a video could make them more willing to participate. They felt that the video created a feeling of connectedness with the researchers from the beginning of the study. One mother expressed it this way: “It is harder to say no face to face, even though it is through a screen” [mother 8]. The mothers felt that the researcher was there in the room with them even though it was through a video on a screen. Mother 10 expresses this contradiction—the researcher being present while simultaneously at a distance—in this quotation: “[the video] works well in that way that it is personal, but without it being face-to-face and that you feel that you need to make a decision right away”.

#### Personification of research

The participants generally expressed comfort in the visual aspect of seeing the person behind the project. They liked that the person in the video was “a real human being that wants to help you and future parents” [mother 11]. Participants also expressed that they found it “trustworthy” that the researchers were seen in a location that appears to be their office. Some of the mothers commented that they had seen and read the name of the PI several times on emails etc. and liked to see how she looked. One mother said that it was “nice to get a face on the person that has been behind the project” [mother 8]. Other participants said that they “felt safe” after watching the video and that it was comforting to know who processes your personal data. Some mothers mentioned that as a new parent, they felt sensitive about their children and about disclosing private information as expressed by mother 7: “[the information] is something personal and it is a sensitive situation you are in when you need help like this”.

Several of the participants commented that they found the researcher in the video trustworthy and sincere. They appreciated the time and resources that the researchers spent producing the video. They felt that this sent a signal to the participants about the seriousness of the study. Mother 4 expressed it this way: “I get a nice gut feeling that she [the researcher] is actually presenting her own material”. Many of the participants also stated that it signalled dedication and engagement that the researcher responsible for the project presented the project in the video. They felt that this signalled dedication and engagement because it showed that the study was important to the researcher and something that the researcher was proud of doing. One mother expressed it this way: “It says; ‘I want to take responsibility for this, and be the face of this – I am actually proud of what I am doing.’ It gives a positive effect that you want to stand forward with what you have done” [mother 8]. Although mothers generally found the researcher in the video serious, a few did find the researcher a bit ‘boring’. A few of the participants were not aware that the person in the video was the researcher who was responsible for the project. They encouraged the research team to make this aspect clearer in the video.

We asked the participants if it made a difference to them who appeared in the video: a trial researcher or an actor. A few participants felt that this would not make a difference but the vast majority found that it made the study more personal. This was expressed by mother 7 in this quotation: “I feel that she is talking directly to me, and I personally like that better than when you sometimes use animations or cartoons”. A few of the mothers said that it did not make any difference that it was the researcher, but they still appreciated that it was a real person related to the project. Many of the participants thought that seeing the researcher made the project less abstract. Mother 12 said: “I actually like that there is someone behind, that it is not a system that speaks and writes, but a person, who then represents a system”. The participants experience that the researcher appearing in the video explaining the study personifies the study in the minds of the participants and becomes the ‘face’ of the study. This is comforting for the participants and makes the study more trustworthy. Mother 11 expressed it in the following way: “It is not just some big organization where it all ends up in. There are actually some people behind it. I think that often you get a survey […] and you don’t really know where it all ends up”.

#### Retention

Participants expressed that seeing the researcher behind the study in the video creates a familiarity with the researchers and a feeling of connectedness. Mother 8 formulated it this way: “As soon as you see the faces and you establish a relationship, you quickly get a sense of belonging and attachment to the project”. The participants also felt that it adds consistency when they recognize the name of the researcher in e.g. info leaflets, newsletters, and questionnaires. Some of the participants felt that seeing the video created a relationship with the researcher that may enhance the chance of the participants staying in the project over time. Mother 13 expressed this in this quotation: “It will maybe create some kind of relationship […] that you want to continue in the project rather than drop out”.

## Discussion

This study aimed to explore attitudes towards the use of informational ‘talking head’ videos to explain the background, aims, and processes of a trial from the perspectives of parents who participated in two trials of parenting interventions (PMTO and IYPB). Based on a thematic analysis of 24 semi-structured interviews, we identified three overarching themes: (1) general impression of the video, (2) thoughts on participation in research, and (3) recruitment and retention. We found that the participants were positive about using a video for recruitment and that it can contribute to ensuring that all participants understand what they are consenting to. Furthermore, we find that use of videos may not improve recruitment but could potentially improve retention due to the perceived formation of a relationship with the research team.

### Use of video in recruitment

Using an informational video when recruiting for a trial is an easy and accessible way to convey information to the participants. People acquire information in different ways, and it is therefore important to implement a variety of strategies to support recruitment and informed consent procedures, thus ideally combining oral, written, and video information about a trial. Previous research has found that one-size-fits-all traditional PIS can be a challenge especially for participants with a low education level or poor literacy or numeracy abilities [17, 22]. Digital platforms are increasingly being used for recruitment. Participants of low socioeconomic backgrounds do have a high interest in using digital platforms but may require additional upfront human support to be able to gain access [37].

Using a video in the recruitment process can be an advantage specifically for these vulnerable groups of participants that are important to include in trials but may prefer video material to the written material. Low literacy affects around 750 million adults worldwide and can limit a person’s ability to acquire key information and process and recall complex information [36]. A recent review of the use of visual aids in health education materials for persons with low literacy levels supports the use of videos as they find that pictograms and videos were the most effective visual aids [36]. However, the review also highlighted large gaps in the research base and acknowledge that what works well for persons with low literacy may not work for everyone. It may therefore be necessary to tailor the information to different groups of participants.

When using videos, there is a fine balance between keeping an appropriate length of the video while simultaneously providing enough information. If more information than what can be contained in a short video needs to be provided, it may be necessary to create e.g. a website with additional information. Mattock et al. suggest that several shorter videos (e.g. 30 s each) addressing different topics such as assessment, consent, and randomization may be optimal [26]. This is likely of particular importance for multisite or more complex trials.

To ensure that participants get the best impression of the research project that they are invited to participate in, the video must be of high quality. It is relatively easy to produce a video but if it does not look professional, it may send the wrong signals to the participants. Technology has changed markedly since 2012 when we recorded the videos and they would probably look different if we made them today. One thing that for example has become very common is adding subtitles to videos so they can be accessed without the sound on. Many research projects apply a participatory design where participants are included in a qualitative preliminary phase to explore the needs and preferences of the potential participants and the best possible way of targeting them [3, 38]. Studies find that participatory designs can lead to significantly improved PIS [16] and could therefore be important when preparing PIS and other recruitment materials such as recruitment videos in future trials. When applying a participatory design, it is however important to design it thoughtfully to avoid unintended consequences such as further marginalization and adding a burden to under-represented and/or over-researched populations [39].

### Ethics

Approximately half of the trial participants do not read the consent document carefully [22]. Ethically, participants must understand the consequences of participating in a trial. To establish informed consent, five concepts must be considered: voluntariness, capacity, disclosure, understanding, and decision [21]. We do not know if our participants just forgot about the information materials, if they did not understand it, or if they did not receive the materials. Links to the recruitment videos were exclusively shared through email and text within the trials when they were recruiting so we are relatively sure that a substantial number of participants did access the trial video. An important recommendation for future trials using video information is therefore to make sure that the video is easily accessible and it is clear for the recruiting staff that watching the video before consent is mandatory. Making it mandatory could however have a negative effect on recruitment if parents do not like the video.

Although it is common for trial participants to forget or not understand the information within PIS [18–20], it is crucial from an ethical standpoint that participants understand if they are consenting to a trial with randomization. Randomization is a component of trials that is particularly difficult for participants to understand—only around 50% of participants have an adequate understanding of the concept [40]. For more vulnerable groups of participants, e.g. with a lower level of education and a low level of literacy, randomization is particularly difficult to understand [21]. Aversion to randomization is also a common reason for declining to participate [41]. For participants with low socioeconomic background, studies have found that it can be helpful to use standard metaphors, such as ‘the toss of a coin’, or culturally derived metaphors, such as ‘sex of a baby’ when explaining the randomization process [41].

Combining text, audio, and graphic material is increasingly used in recruitment materials. Combining more than one mode (such as text and graphic) and giving information across more than one cognitive channel (such as both audio and visual) can contribute to increased comprehension and a deeper understanding as it provides potential participants with the possibility of acquiring information more than once using different modalities [42]. Adding a recruitment video to the standard written material can thereby contribute to reducing misunderstandings and ‘non-informed’ consent [21]. With the highly publicized introduction of the General Data Protection Regulation (GDPR) in Europe, trial participants have become more aware of protecting their data. Several mothers expressed appreciation about seeing the researcher who would be responsible for safeguarding their personal and sensitive data in the video. The need for participant information may have increased since the introduction of GDPR. As video may be optimal to convey important information to some groups of participants in an easily accessible format, the use of video may be useful in ensuring recruitment processes comply with GDPR. Applying a video is in line with the newest guidelines from the UK Medical Research Council (MRC) on consent and the preparation of information for participants stating, “If you are consenting people who cannot read, the Participant Information Sheet may be read to the potential participant as a ‘script’. Alternatively, consider using alternative formats to convey the information for example images, diagrams, audio, video, or online materials” [43].

Our participants were mainly motivated by altruism, but some also wanted to access help. This is in line with several other studies finding that the engagement of the participants most often can be characterized as either banal altruism (where participants participate to contribute positively to society) or conditional altruism (where participants recognize that trial participation can benefit themselves) [44–46]. To feel connected to and care for others is a basic human need, and a video may act as a medium to activate altruistic feelings. These motivational factors represent key issues that should be kept in mind when developing recruitment videos for future projects recruiting mothers of young children.

### Recruitment and retention

When recruiting vulnerable groups for trials, it is common that ethics committees or frontline staff act as gatekeepers who want to protect participants from participating in trials [47]. However, vulnerable groups of participants often report that participation in a trial was worth it [47]. Some of our participants felt proud to be part of a study after watching the video. If participants feel proud about participating in a trial, this can contribute to the feeling of commitment to the study and improve retention rates. It is, however, also possible, that some participants feel less committed to a study after watching a video if e.g. the person in the video has particular characteristics that the viewer is biased against.

Having the researchers behind the trial perform in the video provides a medium for ‘humanizing’ the trial [48, 49]. By seeing the researcher in person and hearing her voice, the researcher behind the trial, the study becomes less abstract and provides the participant with a feeling of a personal connection to the researcher without having met face-to-face. If participants feel more committed to the researcher and the project after watching a video, this may contribute to improved recruitment and retention rates. Trial representatives such as health visitors and social workers who meet the participants play a central role in the building of trust and commitment [50] and can ultimately influence participants’ decision-making regarding participation. Regardless of how professional and personal the informational video appears, staff at the frontline of the study whom participants do meet face-to-face also need to engender similar qualities of trust and commitment. Making it possible for participants to meet frontline staff face-to-face and study staff in a talking head video could further add to trust-building.

Using a video for recruitment seems to be beneficial in enhancing the understanding of what participation in trials means for the participant. However, it may not necessarily translate into increased recruitment [26]. Indeed, a better understanding of what participation in a trial involves concerning e.g. randomization or time commitment may lead to fewer participants enrolling [26]. It is possible that the greatest potential for use of videos in recruitment is concerning retention. A recent review found that multimedia information appears to improve long-term knowledge retention rates [51]. A video can provide important information to participants who do not prefer traditional PIS such as participants with a low education level, poor literacy, or numeracy abilities. A video can also contribute to trust-building and may make participants more committed to the study. This can likely improve retention rates. Further research is needed to examine the effectiveness of informational videos concerning increasing recruitment and especially retention to trials.

### Limitations

A limitation of this study is that the participants were asked to comment retrospectively, and in many cases hypothetically, about the informational ‘talking head’ video that was available to them at the time they were recruited to each trial. Although this study includes both a clinical and a universal sample, the participants for this study constitute a convenience sample and there may be an element of selection bias in the views represented. The participants who chose to take part in the interviews after completing the trials may be extraordinarily engaged in research and might not be able to provide information about why others would not want to participate in a study that they actively chose to participate in. Another limitation of the study is the lack of co-design (or participatory design) methods in the production of the video, i.e. that parents were not asked what they would like to see or the information they would need to make an informed decision before the development of the video. An example of best practice with regard to co-design can be observed in the TRials Engagement in Children and Adolescents (TRECA) trial [52, 53]; the study team implemented a consultation process with end-users of the videos drawing on interview and focus group methods. In the first round of consultation, end-users worked with the research team to discuss and prioritize information needs (based on needs identified in previous research [52, 53]), explored topics that would work best in animated form, and consulted on the design features of the videos such as characters and colour palettes. In the second round, the group reviewed the prototype videos and gave constructive feedback on how to improve them.

Further robust research is needed with larger samples on the effectiveness of different recruitment strategies including videos and multimedia information resources (MMIs). As outlined by Treweek et al. [5], a rigorous test of such methods is a ‘study within a trial’ (SWAT), i.e. an RCT study that compares one method with one or more alternative recruitment methods, nested in a ‘host trial’. In addition to being randomly allocated to a treatment or intervention, a participant recruited to the ongoing host trial would also be randomized to a recruitment method.

## Conclusions

The use of video for recruitment in trials is acceptable for the participants and is a useful supplement to written PIS as the different formats address different needs. For trials aimed at vulnerable groups characterized by e.g. low literacy, children with learning disabilities, or parents with mental health problems, adding a video to the recruitment may be critical. It is possible that an informational video may not improve recruitment, but our results indicate that a professionally produced video featuring a key member of the study team produced a feeling of commitment to the study that could impact retention rates. Key recommendations are to use a participatory design when developing the video, to ensure a ‘professional’ look to the video, to supplement videos with paper-based information, to keep the length to < 3 min, and for the ‘talking head’ part to be played by a key member of the study team. We believe that this knowledge can be transferred to recruitment in a non-trial context to support face-to-face recruitment in usual services.

The use of videos may be complicated if they are not compatible with the expectations of ethics committees, research funders, or gatekeepers of secondary datasets. As more evidence is generated about the potential effectiveness of videos and other forms of multimedia information resources, researchers will need to ensure dissemination efforts reach these key audiences. As evidenced by increasing demands for trial registration and open access publications, there has been a significant shift in recent years towards making research transparent and more accessible to the public [54]. The production of informational videos for trials may serve to contribute to that agenda by increasing opportunities for concise and informative dissemination to wider audiences and in different mediums.

## Supplementary Information

**Additional file 1:** Interview guide

## Data Availability

Audio files or transcriptions are available by request from the corresponding author.

## Abbreviations

PIS: Participant information sheet
IYPB: Incredible Years Parents and Babies
PMTO: Parent Management Training Oregon
PI: Principal investigator
ADHD: Attention deficit hyperactivity disorder
GDPR: General Data Protection Regulation
MRC: Medical Research Council
TRECA: TRials Engagement in Children and Adolescents
MMI: Multimedia information resource
SWAT: Study within a trial
NIHR: National Institute for Health Research

## References

[1] McDonald AM, Knight RC, Campbell MK, Entwistle VA, Grant AM, Cook JA (2006). What influences recruitment to randomised controlled trials? A review of trials funded by two UK funding agencies. Trials..

[2] Bower P, Wilson S, Mathers N. Short report: How often do UK primary care trials face recruitment delays? 2007;601–603. 10.1093/fampra/cmm051 17872907

[3] Martin-Kerry J, Bower P, Young B, Graffy J, Sheridan R, Watt I (2017). Developing and evaluating multimedia information resources to improve engagement of children, adolescents, and their parents with trials (TRECA study): study protocol for a series of linked randomised controlled trials. Trials.

[4] Haidich AB, Ioannidis JPA (2001). Patterns of patient enrollment in randomized controlled trials. J Clin Epidemiol.

[5] Treweek S, Mitchell E, Pithkethly M, Kjeldstrom M, Taskila T, Johansen M, et al. Strategies to improve recruitment to randomised controlled trials. Cochrane Database Syst Rev. 2010;1. 10.1002/14651858.MR000013.pub5. 10.1002/14651858.MR000013.pub4 20091668

[6] Britton A, McKee M, Black N, McPherson K, Sanderson C, Bain C (1999). Threats to applicability of randomised trials: exclusions and selective participation. J Health Serv Res Policy. SAGE Pub.

[7] Treweek S, Lockhart P, Pitkethly M, Cook JA, Kjeldstrøm M, Johansen M, Taskila TK, Sullivan FM, Wilson S, Jackson C, Jones R, Mitchell ED (2013). Methods to improve recruitment to randomised controlled trials: Cochrane systematic review and meta-analysis. BMJ Open.

[8] Stateva M, Minton J, Beckett C, Doolan M, Ford T, Kallitsoglou A, Scott S (2012). Challenges recruiting families with children at risk of anti-social behaviour into intervention trials: lessons from the Helping Children Achieve (HCA) study. J Child Serv [Internet].

[9] Woolfall K, Shilling V, Hickey H, Smyth RL, Sowden E, Williamson PR, et al. Parents’ agendas in paediatric clinical trial recruitment are different from researchers’ and often remain unvoiced: a qualitative study. PLoS One. 2013;8(7). 10.1371/journal.pone.0067352. 10.1371/journal.pone.0067352 PMC3701006 23844006

[10] Hoberman A, Shaikh N, Bhatnagar S, Haralam MA, Kearney DH, Colborn DK, Kienholz ML, Wang L, Bunker CH, Keren R, Carpenter MA, Greenfield SP, Pohl HG, Mathews R, Moxey-Mims M, Chesney RW (2013). What factors influence parental decisions to participate in clinical research: consenters versus non-consenters. JAMA Pediatr.

[11] Varma S, Jenkins T, Wendler D (2008). How do children and parents make decisions about pediatric clinical research?. J Pediatr Hematol Oncol.

[12] Caldwell PHY, Dans L, De Vries MC, Newman J, Sammons H, Spriggs M (2012). Standard 1: consent and recruitment. Pediatrics..

[13] Tarnowski KJ, Allen DM, Mayhall C, Kelly PA. Readability of pediatric biomedical research informed consent forms. Pediatrics. 1990;85:58 – 62. 2296494

[14] Ogloff JRP, Otto RK (1991). Are research participants truly informed? Readability of informed consent forms used in research. Ethics Behav.

[15] Eder ML, Yamokoski AD, Wittmann PW, Kodish ED. Improving informed consent: suggestions from parents of children with leukemia. Pediatrics. 2007, 119:e849–59. 10.1542/peds.2006-2208 17403829

[16] Knapp P, Raynor DK, Silcock J, Parkinson B. Can user testing of a clinical trial patient information sheet make it fit-for-purpose? - a randomized controlled trial. BMC Med. 2011;9(1). 10.1186/1741-7015-9-89. 10.1186/1741-7015-9-89 PMC3152894 21777435

[17] Griffin JM, Struve JK, Collins D, Liu A, Nelson DB, Bloomfield HE (2006). Long term clinical trials: how much information do participants retain from the informed consent process?. Contemp Clin Trials.

[18] Stead M, Eadie D, Gordon D, Angus K (2005). “Hello, hello - It’s English I speak!”: a qualitative exploration of patients’ understanding of the science of clinical trials. J Med Ethics.

[19] Tait AR, Voepel-Lewis T, Levine R (2015). Using digital multimedia to improve parents’ and children’s understanding of clinical trials. Arch Dis Child.

[20] Fortun P, West J, Chalkley L, Shonde A, Hawkey C (2008). Recall of informed consent information by healthy volunteers in clinical trials. Qjm..

[21] Tam NT, Huy NT, Thoa LTB, Long NP, Trang NTH, Hirayama K, Karbwang J (2015). Participants’ understanding of informed consent in clinical trials over three decades: systematic review and meta-analysis. Bull World Health Organ.

[22] Tait AR, Voepel-Lewis T (2015). Digital multimedia: a new approach for informed consent?. JAMA..

[23] Little M, Berry V, Morpeth L, Blower S, Axford N, Taylor R (2012). The impact of three evidence-based programmes delivered in public systems in Birmingham, UK. Int J Confl Violence.

[24] Morpeth L, Blower S, Tobin K, Taylor RS, Bywater T, Edwards RT, Axford N, Lehtonen M, Jones C, Berry V (2017). The effectiveness of the Incredible Years pre-school parenting programme in the United Kingdom: a pragmatic randomised controlled trial. Child Care Pract.

[25] Winslow EB, Braver S, Cialdini R, Sandler I, Betkowski J, Tein JY, Hita L, Bapat M, Wheeler L, Lopez M (2018). Video-based approach to engaging parents into a preventive parenting intervention for divorcing families: results of a randomized controlled trial. Prev Sci.

[26] Mattock HC, Ryan R, O’Farrelly C, Babalis D, Ramchandani PG (2020). Does a video clip enhance recruitment into a parenting trial? Learnings from a study within a trial. Trials.

[27] Terblanche M, Burgess L (2010). Examining the readability of patient-informed consent forms. Open Access J Clin Trials.

[28] Pontoppidan M (2015). The effectiveness of the Incredible Years^TM^ Parents and Babies Program as a universal prevention intervention for parents of infants in Denmark: study protocol for a pilot randomized controlled trial. Trials.

[29] Pontoppidan M, Klest SK, Sandoy TM (2016). The Incredible Years Parents and Babies program: a pilot randomized controlled trial. PLoS One.

[30] Pontoppidan M, Sandoy TM, Klest SK (2020). One-year follow-up of The Incredible Years Parents and Babies Program: a pilot randomized controlled trial. Scand J Child Adolesc Psychiatry Psychol.

[31] Lindberg MR, Hansen H, Scavenius C (2017). Midtvejsevaluering af bedre familiebehandling. Et randomiseret kontrolleret forsøg med Parent Management Training – Oregon og anden familiebehandling.

[32] Lindberg MR, Molberg MR, Scavenius C (2019). Effekten af familiebehandling i Danmark: Et felteksperiment med Parent Management Training–Oregon (PMTO) og anden familiebehandling. VIVE-Det Nationale Forsknings-og Analysecenter for Velfærd.

[33] Novick G (2008). Is there a bias against telephone interviews in qualitative research?. Res Nurs Health.

[34] Carr ECJ, Worth A (2001). The use of the telephone interview for research. NT Res.

[35] Braun V, Clarke V (2006). Using thematic analysis in psychology. Qual Res Psychol.

[36] Mbanda N, Dada S, Bastable K, Ingalill GB, Ralf W (2021). S. A scoping review of the use of visual aids in health education materials for persons with low-literacy levels. Patient Educ Couns.

[37] Hernandez-Ramos R, Aguilera A, Garcia F, Miramontes-Gomez J, Pathak LE, Figueroa CA, Lyles CR (2021). Conducting internet-based visits for onboarding populations with limited digital literacy to an mhealth intervention: development of a patient-centered approach. JMIR Form Res.

[38] Sheridan R, Martin-Kerry J, Watt I, Higgins S, Stones SR, Taylor DH, et al. User testing digital, multimedia information to inform children, adolescents and their parents about healthcare trials. J Child Heal Care. 2018; 10.1177/1367493518807325 30384772

[39] Ní Shé É, Harrison R (2021). Mitigating unintended consequences of co-design in health care. Health Expect.

[40] Falagas ME, Korbila IP, Giannopoulou KP, Kondilis BK, Peppas G. Informed consent: how much and what do patients understand? Am J Surg. 2009, 198:420–35 Available from: 10.1016/j.amjsurg.2009.02.010. 10.1016/j.amjsurg.2009.02.010 19716887

[41] Krieger JL, Parrott RL, Nussbaum JF (2011). Metaphor use and health literacy: a pilot study of strategies to explain randomization in cancer clinical trials. J Health Commun.

[42] Hughson JA, Woodward-Kron R, Parker A, Hajek J, Bresin A, Knoch U, et al. A review of approaches to improve participation of culturally and linguistically diverse populations in clinical trials. Trials. 2016;17:1–10. Available from: 10.1186/s13063-016-1384-3 10.1186/s13063-016-1384-3 PMC4880985 27229153

[43] NHS Health Research Authority. Informing participants and seeking consent. 2019. Available from: https://www.hra.nhs.uk/planning-and-improving-research/best-practice/informing-participants-and-seeking-consent/. Accessed 6 Jan 2021.

[44] McCann SK, Campbell MK, Entwistle VA (2010). Reasons for participating in randomised controlled trials: conditional altruism and considerations for self. Trials..

[45] Carrera JS, Brown P, Brody JG, Morello-Frosch R. Research altruism as motivation for participation in community-centered environmental health research. Soc Sci Med. 2018;196:175–81 Available from: 10.1016/j.socscimed.2017.11.028. 10.1016/j.socscimed.2017.11.028 PMC5768448 29190538

[46] Phipps FM, Price AD, Ackers-Johnson J, Cook PA, Lythgoe J. Part 1: a qualitative description of participation in an eight-week infant skin integrity study. Br J Midwifery. 2021;29(4):200–7. 10.12968/bjom.2021.29.4.200.

[47] Alexander S, Pillay R, Smith B (2018). A systematic review of the experiences of vulnerable people participating in research on sensitive topics. Int J Nurs Stud.

[48] Haan M, Ongena YP, Vannieuwenhuyze JTA, De Glopper K (2017). Response behavior in a Video-Web Survey: a mode comparison study. J Surv Stat Methodol.

[49] Pedersen MJ, Bojesen AB, Rayce SB, Pontoppidan M. Using informational video to elicit participation in online survey research: a randomized controlled trial. Int J Public Opin Res. 2020. Available from: 10.1093/ijpor/edaa023.

[50] Galli L, Knight R, Robertson S, Hoile E, Oladapo O, Francis D, Free C (2014). Using marketing theory to inform strategies for recruitment: a recruitment optimisation model and the txt2stop experience. Trials..

[51] Nishimura A, Carey J, Erwin PJ, Tilburt JC, Murad MH, McCormick JB. Improving understanding in the research informed consent process: a systematic review of 54 interventions tested in randomized control trials. BMC Med Ethics. 2013;14(1):28. 10.1186/1472-6939-14-28. 10.1186/1472-6939-14-28 PMC3733934 23879694

[52] Kirkby HM, Calvert M, Draper H, Keeley T, Wilson S (2012). What potential research participants want to know about research: a systematic review. BMJ Open.

[53] Luchtenberg M, Maeckelberghe E, Locock L, Powell L, Verhagen AAE (2015). Young people’s experiences of participation in clinical trials: reasons for taking part. Am J Bioeth.

[54] Goldacre B, Gray J (2016). OpenTrials: towards a collaborative open database of all available information on all clinical trials. Trials.

