# Supplementary material for: What do parents think of using informational videos to support recruitment for parenting trials? A qualitative study
Source: Trials. 2021 Dec 4;22:872. doi: 10.1186/s13063-021-05826-0 (PMC8642858; doi:10.1186/s13063-021-05826-0)
Supplement: Supplementary file 1 — Additional file 1: Interview guide [file 13063_2021_5826_MOESM1_ESM.pdf]

Interviewguide

Da brugen af en video som rekrutterings form er ny. Ønskes der viden om i hvilket omfang den har haft betydning for deltagernes beslutning om deltagelse.	The use of video as recruitment method is new and therefore we wish is to generate knowledge about to what extent it has affected the participants' decision on participation.
Har du set videoen?	Did you watch the video?
Hvad synes du om videoen?<br>• Synes du at videoen indeholdt relevant information?<br>• Fik du den information, som du havde brug for, eller havde du yderligere spørgsmål efter at have set videoen?<br>• Var længden på videoen passende eller synes du, at den var for kort eller for lang?<br>• Har du forslag til, hvordan videoen kan gøres bedre?	What do you think about the video?<br>• Do you think that the video contains relevant information?<br>• Did you get the information that you needed - or did you have further questions after you watched the video?<br>• Was the length of the video appropriate or did you think it was too short or too long?<br>• Do you have any suggestions as to how the video can be improved?
Hvad tænker du om det at få informationer via en video som supplement til den skrevne pjece?<br>• Hvilke fordele ser du ved at bruge en video?<br>• Hvilke ulemper ser du ved at bruge en video?<br>• Synes du, at det er en god ide at anvende video som informationskilde en anden gang?	What do you think about getting information via a video in addition to a written leaflet?<br>• What benefits can you point to when using a video?<br>• What disadvantages can you point to when using a video?<br>• Do you think that it is a good idea to use video as a source of information in a later project?
Gjorde det en forskel for dig at se forskeren på filmen?<br>• Tænker du at det har haft en betydning for din deltagelse?	Did it make a difference for you to see the researcher appear on the video?<br>• Do you think that it had an impact on your participation?
Hvilke tanker gjorde du dig omkring det at skulle deltage i et forskningsprojekt, inden du sagde ja?<br>• Havde du nogle bekymringer<br>• Hjalp videoen ift. Nogle af disse tanker eller bekymringer?	What kind of thoughts did you have about participating in a research project?<br>• Did you have any worries?<br>• Did the video help with regard to any of these thoughts or worries?
Hvorfor ville du gerne deltage i forskningsprojektet?<br>• Gjorde videoen, at du fik mere lyst til at deltage?<br>• Gjorde det at du kunne se forskeren på videoen, at du fik mere lyst til at deltage?<br>• Tror du, at du ville have sagt ja til at deltage, hvis du kun havde læst pjecen og ikke havde set videoen?<br>• Eller: tror du, at du ville have sagt ja til at deltage, hvis du kun havde set videoen og ikke læst pjecen?	Why did you want to participate in the research project?<br>• Did you feel more enthusiastic about participating after having seen the video?<br>• Did the fact that you could see the researcher in the video make you feel more like participating?<br>• Do you think that you would have participated if you had only read the leaflet and not seen the video?<br>• Or: Do you think that you would have participated if you only had seen the video and not read the leaflet?
